# Preparation of a robust hierarchical superhydrophobic surface with superior corrosion resistance

**DOI:** 10.1039/d5ra02098k

**Published:** 2025-07-07

**Authors:** Mengqing Li, LiangJun Song, Yu Zheng, Nuoran Liu, Huizhu Yu, Rencheng Jin, Tengfei Xiang, Ruiqian Li

**Affiliations:** a Institute of Noise & Vibration, Naval University of Engineering Wuhan 430033 China; b School of Chemistry & Materials Engineering, Fuyang Normal University Fuyang 236037 China liruiqian2008@163.com; c School of Civil Engineering and Architecture, Anhui University of Technology Ma'anshan 243002 China xiangtf@ahut.edu.cn

## Abstract

The design and facile fabrication of a hierarchical superhydrophobic surface with robust stability is the key for industrialization of superhydrophobic materials. Herein, a robust hierarchical superhydrophobic Zn coating (EAE-Zn) with armor-like structure was fabricated by two-step electrodeposition accompanied with an indispensable intermediate activation. The results showed that the activation treatment was favorable for the formation and growth of nano-flakes on the side surfaces of vertically aligned micro-flakes. Notably, the armor-like EAE-Zn coating still sustained its water repellency even after suffering from sandpaper abrasion, tape peeling, water drop impact and long-term immersion in salt solution, indicating that the vertically aligned micro-plates acting as “armor” could prevent effectively the abrasion of the fragile nano-structures. Besides, the superior non-wettability and impermeability endowed the EAE-Zn coating with long-term corrosion resistance ability. Therefore, these findings offer a novel strategy to design a hierarchical superhydrophobic surface with robust stability and superior corrosion resistance.

## Abstract 2

An armour-like hierarchical structured Zn coating is fabricated by two-step electrodeposition combined with an intermediate pre-activation, which shows robust superhydrophobicity and excellent long-term corrosion resistance.

## Graphical abstract

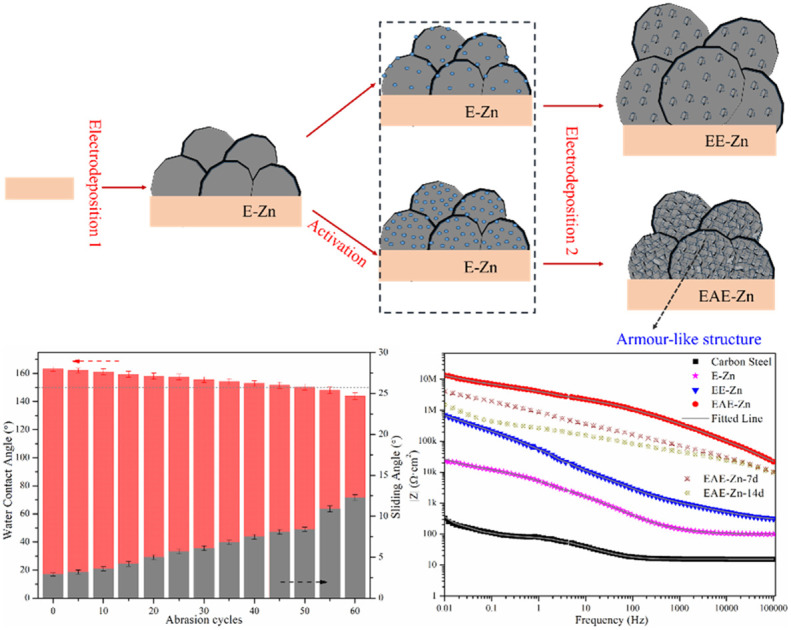

## Introduction

Superhydrophobic surfaces with micro-nano hierarchical structures have attracted unprecedented attention owing to their promising potential applications, such as anti-corrosion,^1–5^ self-cleaning,^6–9^ oil/water separation,^10–13^ anti-fouling,^14–17^ anti-icing,^18–21^ wearable devices,^22–25^ biomedicine,^26–29^ photocatalytic degradation^30–32^*etc.* The construction of robust micro–nano hierarchical structures is a prerequisite for the industrialization of superhydrophobic materials. Although plenty of superhydrophobic surfaces with diverse micro–nano structures have been designed and fabricated, unfortunately, their superhydrophobicity is unsatisfactory because the nano-scale structures are destroyed easily after suffering from mechanical deformation or damage. In recent years, considerable efforts have been devoted to improving the mechanical stability of the superhydrophobic micro–nano hierarchical structures.^33–37^ Generally, there are two main strategies to build robust superhydrophobic surfaces, including structure regeneration and introduction of rigid materials. From the viewpoint of structure regeneration, Wang *et al.*^38^ reported a self-healing superhydrophobic coating with mechanical durability by dripping a mixture of hierarchically structured particles (ACNTB-SiO_2_-KH570) and epoxy, in which the damaged surface was repaired by the coordination of the decomposition of epoxy polymers and the migration/assembly of SiO_2_ particles. Based on the strategy of introducing more rigid materials, Chen *et al.*^39^ constructed circular silica islands on the rubber surface to obtain a robust superhydrophobic surfaces because the periodic array hard silica can effectively protect the soft silicone rubber from external mechanical damage. Despite the fact that great progress has been made in developing robust superhydrophobic surfaces by above-mentioned strategies, it is irrefutable that there still exists limitations and shortcomings.^35^ For example, the superhydrophobic surface composed of rigid materials could be destroyed once the wear exceeds the limit. The self-healing superhydrophobic materials have strict demands for the mobility and long-term stability of bulk materials.

As we all known, the nano-scale structure serving as stressed part sustains high local pressure even under small mechanical load, explaining the poor mechanical stability of superhydrophobic surfaces. Therefore, a series of armored structures with micro structure acting as stressed part and nano structure acting as superhydrophobic part have been designed to improve the mechanical robustness.^40–44^ For example, Deng's^43^ team created an armored micro–nano structure with the micro-scale frame acting as protective armor which could distribute the mechanical pressure and prevent the removal of inside nano-scale structure, leading to robust superhydrophobicity even after harsh mechanical stability test. However, although the “armor structure strategy” provides a new way for preparation of mechanically robust superhydrophobic surface, the preparing process is complicated. Therefore, it has great potential value to fabricate robust and durable superhydrophobic surface in a facile manner for industrialized application. Electrodeposition technology has been widely used in the preparation of superhydrophobic coating due to its advantage of convenient operation, easy control, high efficiency, low cost and diverse deposition parameters, *etc.* The construction of hierarchical superhydrophobic surface is usually conducting by adjusting the processing parameters,^45–49^ such as deposition voltage, deposition time and solution composition. Surface activation is a very important pretreatment during the processing of electroplating. However, to the best of our knowledge, the effect of activation treatment on the surface structure is rarely presented.

Herein, a robust hierarchical superhydrophobic Zn coating with armored structure and superior corrosion resistance was fabricated by facile two-step electrodeposition accompanied with an indispensable activation process. The effect of activation treatment on the deposition process was investigated. Firstly, the vertically aligned micro-scale Zn plates were electrodeposited on the Cu substrate. The following activation treatment leaded to the formation of numerous active sites on the side surface of micro-scale Zn plates, which promoted the nucleation/growth of nano-scale Zn plates. Subsequently, countless crisscrossing nano-scale Zn plates were evenly deposited on the side surface of micro-scale Zn plates during the second electrodeposition, and eventually formed an armor-like hierarchical structured coating. The unique hierarchical structure endows the Zn coating with robust superhydrophobicity and excellent corrosion resistance. This work offers a simple and efficient strategy to fabricate robust hierarchical structure of superhydrophobic metal coating.

## Experimental section

### Materials and reagents

Choline chloride (C_5_H_14_ClNO, ChCl, AR) and zinc chloride (ZnCl_2_·6H_2_O, AR) were supplied by Shanghai Macklin Biochemical Co., Ltd. Ethylene glycol (C_2_H_6_O_2_, AR) and ethanol (C_2_H_5_OH, AR) were purchased from Sinopharm Chemical Reagent Co., Ltd. Stearic acid (C_18_H_36_O_2_, AR) and sodium chloride (NaCl) were obtained from Xilong Scientific Co., Ltd.

### Fabrication of superhydrophobic surfaces

The electrolyte was formed through adding 0.20 M ZnCl_2_·6H_2_O to the choline chloride–ethylene glycol deep eutectic solvent (ChCl–EG DES), and stiring at 70 °C. The armor-like hierarchical structured Zn coating was fabricated by a two-step electrodeposition accompanied with surface modification. Firstly, the micro-scale Zn armor composed of vertically aligned plates was deposited on Cu substrate at a current density of 0.8 A dm^−2^ for 60 min. Before the second step electrodeposition, an activation treatment was conducted to form active sites on the side surface of micro-scale Zn plates, which could promote the formation of Zn nucleus. Then, the nano-scale Zn plates were evenly deposited on the side surfaces of micro-scale plate-like armor. Finally, the armor-like hierarchical structured Zn coating was modified by immersing in stearic acid ethanol solution. The aforementioned coating deposited by one-step electrodeposition and two-step electrodeposition without and with activation treatment are denoted hereafter as E-Zn, EE-Zn and EAE-Zn coating, respectively (Table 1).

**Table 1:** The deposition conditions of E-Zn, EE-Zn and EAE-Zn coatings

Specimens	Electrolyte 1		Activation treatment	Electrolyte 2	
	*I* (A dm^−2^)	*t* (min)		*I* (A dm^−2^)	*t* (min)
E-Zn coating	0.8	60	—	—	—
EE-Zn coating	0.8	60	Distilled water, 20 s	1.5	20
EAE-Zn coating	0.8	60	20 wt% HCl, 20 s	1.5	20

### Characterization of as-prepared coatings

The morphologies of the E-Zn, EE-Zn and EAE-Zn coatings were obtained by field emission scanning electron microscopy (FESEM, Carl Zeiss, Sigma 500). The phase analysis of all the coatings were recorded by X-ray diffraction (XRD, Rigaku, D/MAX-2400) with Cu Ka radiation (*k* = 0.15406 nm). The surface functional groups were analyzed by Fourier transform infrared spectroscopy (FTIR, Thermo Scientific, Nicolet iS50). The water contact angle (WCA) and sliding angle (SA) of the coatings was measured by contact angle instrument (Zhongchen, JC2000D1). The water drop impact test of the EAE-Zn coating was assessed by releasing distilled water (about 100 μL) with a rate of 90 drops per min at the height of 30 cm from the coating. The anti-abrasion property of EAE-Zn coating was evaluated by dragging the coating with a load of 100 g on the 600# sandpaper at a rate of 2.0 cm s^−1^ for 20 cm. The adhesion of the EAE-Zn coating was assessed by applying the 3M4910VHB tape with a load 100 g on the coating for 30 s, then peeling off. The potentiodynamic polarization (Tafel) and electrochemical impedance spectroscopy (EIS) were carried out in 3.5 wt% NaCl aqueous solution to evaluate the corrosion behaviors of three coatings. The coating with an exposed area of 1 cm^2^ was used as the working electrode, a saturated calomel electrode (SCE) and a platinum plate was employed as the reference electrode and counter electrode respectively. To obtain a stable open-circuit potential, the coating was immersed in NaCl solution for 30 min before the electrochemical tests. The Tafel curves were recorded between −1.5 V and +0.3 V *versus* OCP at a scan rate of 1 mV s^−1^. The EIS measurements were conducted in the frequency range from 10^5^ to 10^−2^ Hz with an AC amplitude of 10 mV.

## Results and discussion

### Surface morphology and chemical composition

The E-Zn coating deposited *via* one-step electrodeposition exhibited a single-scale “flower-like” structure, as shown in Fig. 1a and a′, which was composed by lots near vertically aligned Zn plates with a dimension of 5–8 μm. While, the EE-Zn coating (Fig. 1b and b′) fabricated by two-step electrodeposition was composed of vertically aligned micro-plates (12–14 μm) and sparsely distributed nano-plates (400–600 nm). Notably, by introducing the activation treatment, an armor-like EAE-Zn coating featured with double-scale vertically aligned micro-plates (7–9 μm) with even distributed nano-plates (800–1000 nm) was obtained (Fig. 1c and c′). The SEM results indicated that the activation treatment had a significant effect on the surface morphology of Zn coating. Clearly, the formation of both EE-Zn coating and EAE-Zn coating during the second step electrodeposition involved two processes, including the growth of micro-plates and formation/growth of nano-plates. As demonstrated in Fig. 1b and c, the number and size change of the micro-plates and nano-plates indicated that the major reaction was transformed from the growth of micro-plates into the formation/growth of nano-plates after the introduction of activation treatment. Moreover, the sides of vertically aligned micro-plates of EAE-Zn coating densely covered with amounts of nano-plates endowed the EAE-Zn coating with a roughness and armor-like structure, which was considered to be unquestionably beneficial to obtain robust superhydrophobicity.

**Fig. 1:**
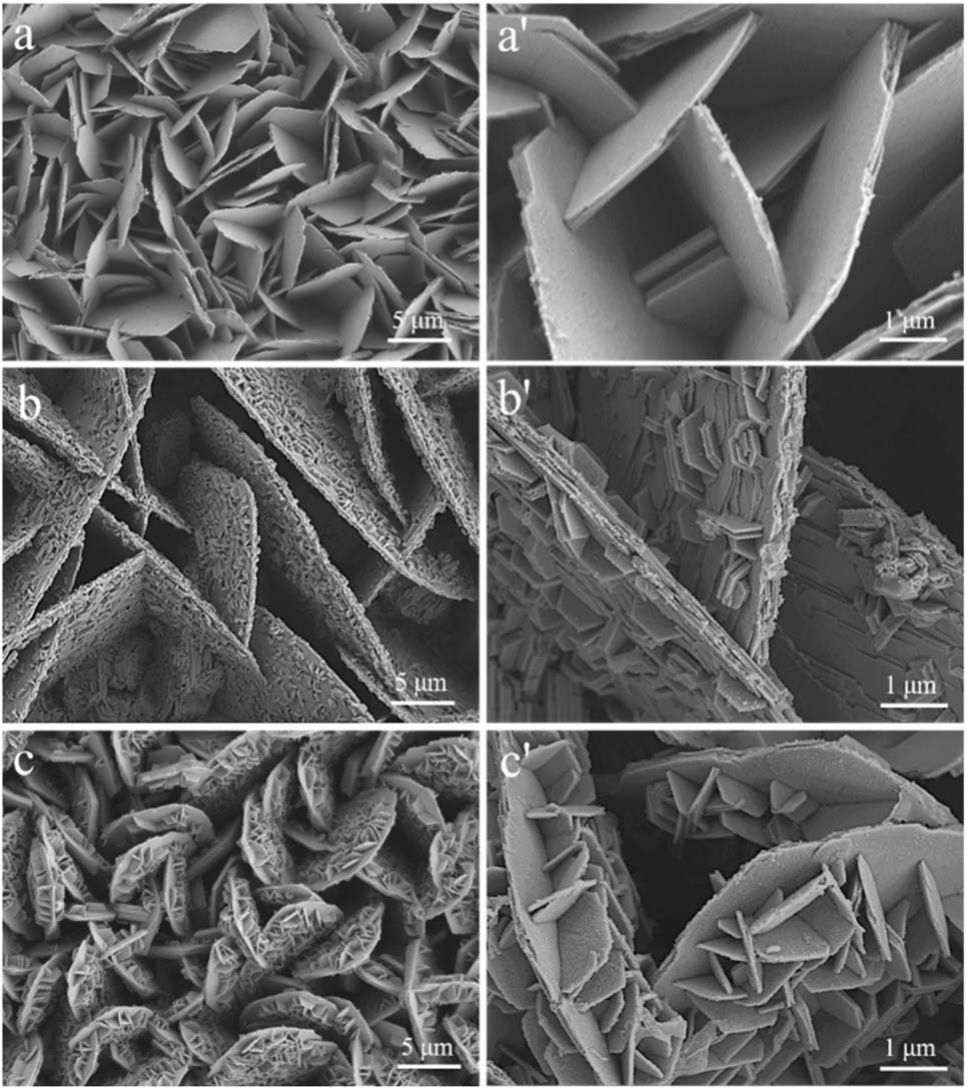
The SEM images of E-Zn (a and a′), EE-Zn (b and b′) and EAE-Zn (c and c′) coatings.

Subsequently, the EDS, FTIR and XRD analyses were implemented to study the composition of the prepared specimens. As shown in Table 2, Zn, C and O elements were all detected from the surface of the E-Zn, EE-Zn and EAE-Zn coatings. The mass ratio of C to O of all the specimens were worthwhile to be noticed, which were closely approximated (about 6 : 1) in stearic acid or zinc stearate. Hence, it could be easily inferred that the hydrophobic functional groups were successfully modified on the three kinds of coatings mentioned above.

**Table 2:** The element composition of E-Zn, EE-Zn and EAE-Zn coatings

Specimens	Zn (wt%)	C (wt%)	O (wt%)
E-Zn coating	92.79	6.15	1.06
EE-Zn coating	89.45	9.02	1.53
EAE-Zn coating	83.73	13.89	2.38

**Fig. 2:**
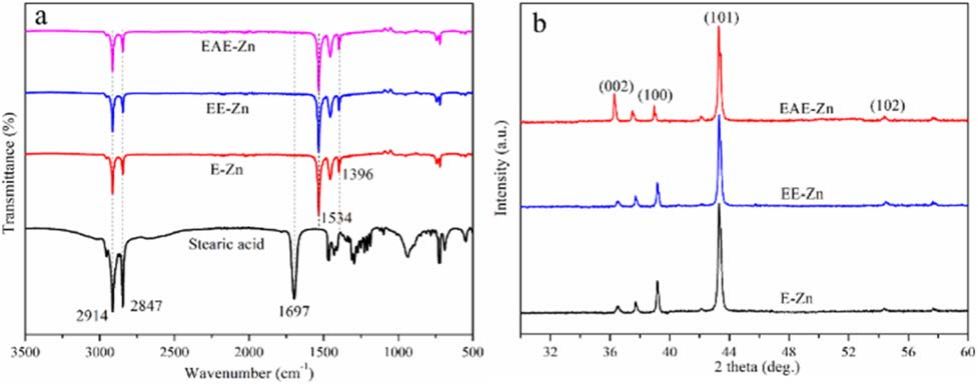
(a) The FTIR spectra of stearic acid, E-Zn, EE-Zn and EAE-Zn coatings; (b) the XRD patterns of E-Zn, EE-Zn and EAE-Zn coatings.

In order to further confirm the components of the low-energy material, the FTIR spectra of stearic acid, E-Zn, EE-Zn and EAE-Zn coatings were shown in Fig. 2a. Undoubtedly, the asymmetrical and symmetrical stretching vibrations of C–H bond of hydrophobic functional groups at 2914 cm^−1^ and 2847 cm^−1^ were detected from three Zn-based coatings as well as stearic acid. The adsorption peak at 1697 cm^−1^ was designated to the –COOH group of stearic acid. Significantly, for the E-Zn, EE-Zn and EAE-Zn coatings, the adsorption peak of –COOH group disappeared, and meanwhile, two strong absorption peaks caused by the –COO^−^ group appeared at 1534 cm^−1^and 1396 cm^−1^.^50^ There was no doubt that the hydrophobic material modified on surface of Zn-based coatings was zinc stearate. In addition, the crystal orientation of three Zn-based coatings were characterized by XRD and shown in Fig. 2b. The diffraction peaks indexed at 2*θ* = 36.3°, 39.0° and 43.3° were attributed to (002), (100) and (101) planes of Zn (JCPDS 04-0831), respectively. It could be found that the diffraction peaks and preferential orientation of E-Zn, EE-Zn and EAE-Zn coatings were identical, indicating the activation treatment had no effect on the phase structures of Zn coating.

### Electrodeposition procedure

Based on the results of SEM, FTIR and XRD, the electrodeposition procedure for three Zn-based coatings was proposed and illustrated as Fig. 3. In the first step electrodeposition, the nucleation and vertical growth of the hexagonal Zn was occurred on the Cu substrate, and eventually obtained a flower-like structured Zn coating composed of plenty of micro-plates with a size of 5–8 μm. Interestingly, the EE-Zn coating with or without activation treatment presented completely different electrodeposition behaviors in second step electrodeposition. In the absence of activation treatment, the electro-crystallization process was dominated by the growth of micro Zn plates deposited in first step electrodeposition, at the same time, a few Zn nuclei was formed and grown on the sides. Finally, an approximately double-scale structure composed of vertically aligned micro plates (12–14 μm) and sparsely distributed nano plates (400–600 nm) was fabricated on the Cu substrate. More importantly, the electro-crystallization process changed from the growth of micro Zn plates mainly to the formation/growth of nano plates mainly after activation treatment. This transformation was attributed to the formation of countless active sites on the side surfaces of EE-Zn coating triggered by activation treatment, providing advantages for the nucleation of Zn. As a result, numerous Zn crystal nucleus was formed on the active surface and developed countless crisscrossing nano-plates. After above processes, an armor-like structured Zn coating composed of vertically aligned micro plates (7–9 μm) and crisscrossing nano-plates (800–1000 nm) was obtained. In the final step, the as-prepared specimens were modified by stearic acid to form an even zinc stearate monolayer on the surface. The unique armor-like hierarchical structure shows great potential in improving the robustness of superhydrophobic surface.

**Fig. 3:**
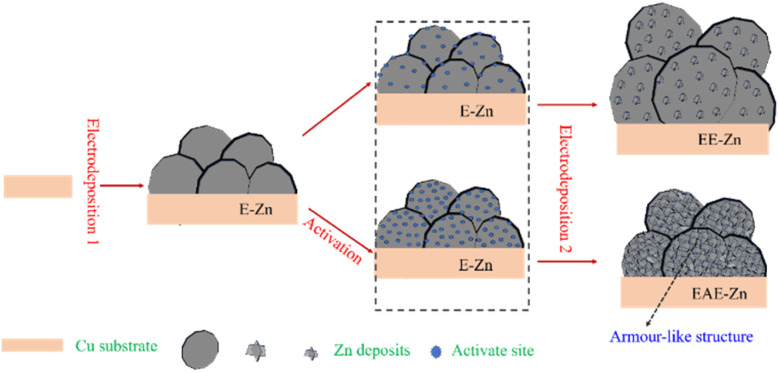
The electrodeposition schematics of E-Zn, EE-Zn and EAE-Zn coatings.

### Surface wetting

**Fig. 4:**
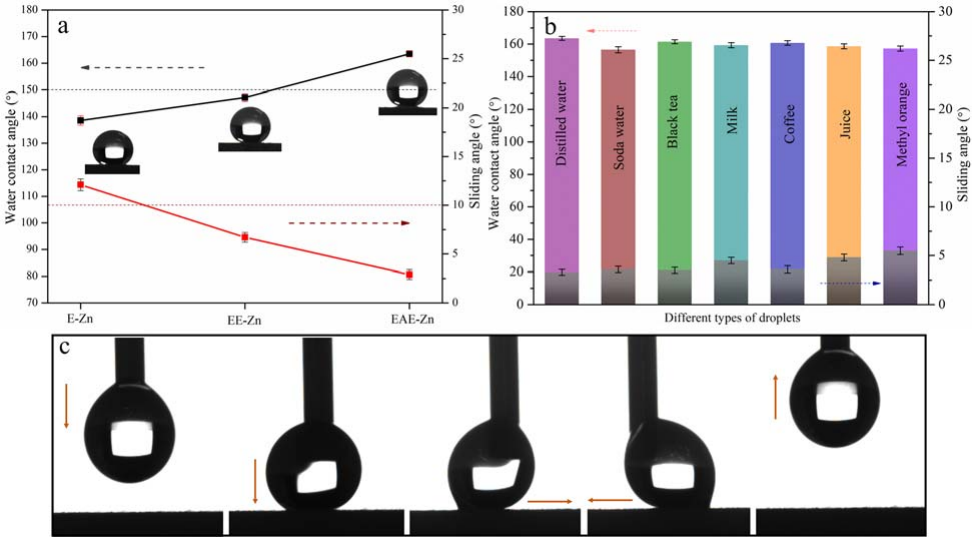
The water contact angle of E-Zn, EE-Zn and EAE-Zn coatings (a); the water contact angle of EAE-Zn coating toward different droplets (b); the dragging test of water droplets on the surface of superhydrophobic EAE-Zn coating (c).

To evaluate the wettability of three Zn coatings, the water contact angle (WCA) and slide contact angle (SA) were measured and studied, as shown in Fig. 4a. As a reference, the WCA of the E-Zn coating with a single-scale flower-like structure was only about 138.6 ± 1.8°. While, owing to the formation of a few nano plates on the surface of flower-like structure, the WCA of the EE-Zn coating increased to 146.3 ± 1.6°. It was noteworthy that the WCA of the EAE-Zn coating was improved to 163.6 ± 1.2° when the surface of flower-like structure was covered completely by numerous crisscrossing nano-plates. In addition, the armor-like structured EAE-Zn coating also exhibited desirable super-repellency to different kinds of droplets such as soda water, black tea, milk, coffee, juice, and methyl orange (Fig. 4b), indicating the superhydrophobic surface possessed a wide suitability for a variety of liquids. Furthermore, the dragging test of water droplets on the surface of superhydrophobic EAE-Zn coating was implemented. As shown in Fig. 4c, a water droplet with a volume of 6 μL was contacted, compressed and dragged on the surface of EAE-Zn until large deformation of the droplet occurred, which could be easily separated from the superhydrophobic surface. This indicated that the armor-like superhydrophobic coating possessed an ultra-low adhesion. Therefore, it could be speculated that the excellent superhydrophobicity and low adhesion property were mainly ascribed to the armor-like hierarchical structure, which could easily trap a large amount of air in the interspaces of micro–nano plates and form a “stable” air isolating layer between the solid surface and liquid.

### Chemical and mechanical stability

**Fig. 5:**
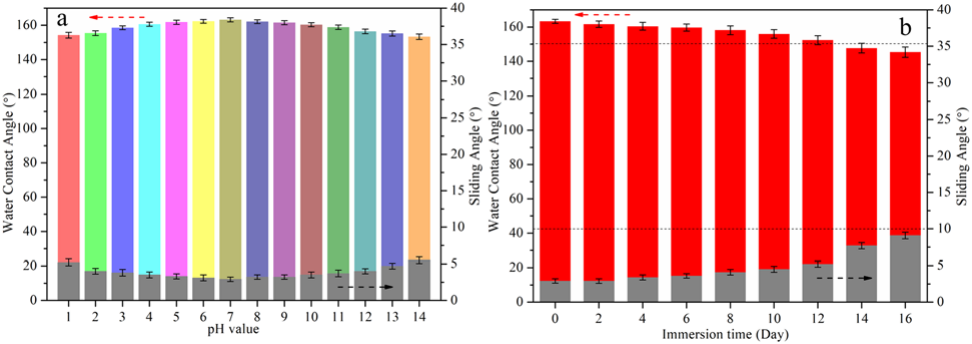
The chemical stability tests of EAE-Zn coating under different harsh environment: different pH value (a) and different immersion time in 3.5 wt% NaCl solution (b).

The durability of superhydrophobic surface is an important index for practical applications. In this section, solutions of 3.5 wt% NaCl with different pH were used to evaluate the chemical stability of the EAE-Zn coating. As shown in Fig. 5a, when the pH value of water droplets changed from 1 to 14, the armor-like structured EAE-Zn coating still remained superhydrophobicity, with water contact angles higher than 153° and sliding angle lower than 7°, suggesting good acid and alkali resistance of the armor-like structured surface. Subsequently, the effect of immersion time in 3.5 wt% NaCl solution on the wettability of EAE-Zn coating was explored. As shown in Fig. 5b, the EAE-Zn coating still kept superhydrophobic state with a WCA of 151.9 ± 1.6° and a SA of 5.2 ± 0.6° up to 12 days. The excellent chemical stability of the EAE-Zn coating demonstrated that a large quantity of air trapped in the armor-like structures could efficiently impede the invasion of corrosive liquid.

**Fig. 6:**
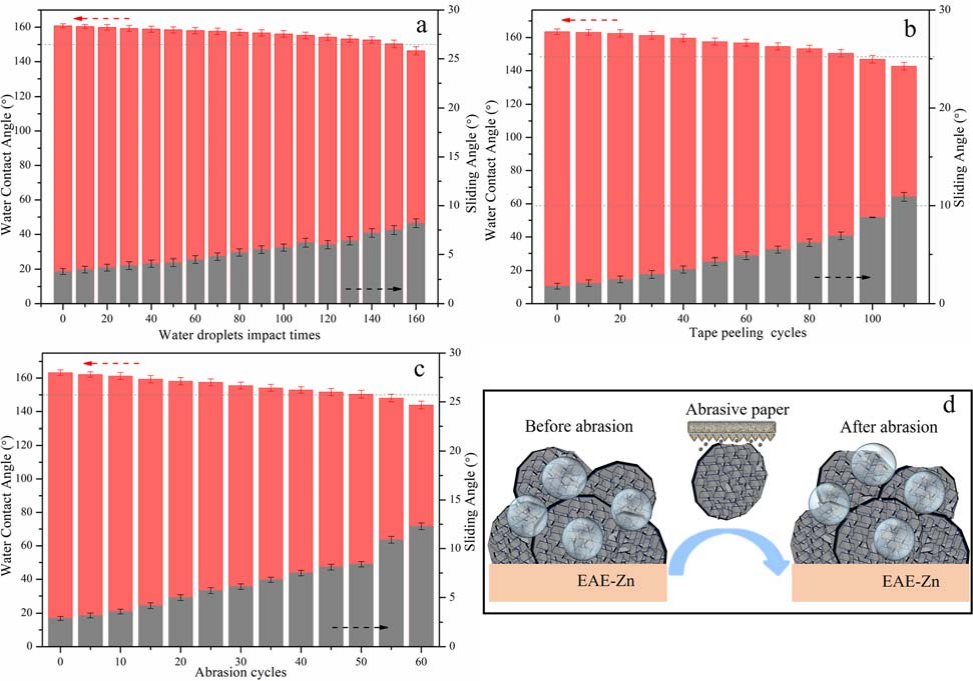
The mechanical stability tests of EAE-Zn coating under different harsh environment: water drop impact test (a), the tape peeling test (b), sandpaper abrasion test (c) and diagram before and after abrasion (d).

Moreover, the water-drop impact test, tape peeling test and sandpaper wear test were implemented to assess the mechanical stability of armor-like structured EAE-Zn coating. It was clear that the armor-like structured EAE-Zn coating retained the superhydrophobicity without substantial changes in WCA and SA when the duration of water dripping was last for 30 min. More impressively, the WCA of the surface was still as high as 150° even after water dripping for 150 min, indicating the armor-like structure had perfect resistance to water droplet impact (Fig. 6a). As shown in Fig. 6b, the EAE-Zn surface still showed stable superhydrophobicity with a WCA of 150.7° and a SA of 7.3° after 90 tape peeling cycles, suggesting the excellent adhesion of the armor-like structure. According to Fig. 6c, the EAE-Zn coating possessed a relatively high WCA of 150.7° and a low SA of 8.7° even suffering from 50 sandpaper abrasion cycles, revealing the armor-like structure presented high resistance to abrasion. Thus, the superior mechanical stability of the armor-like hierarchical structured EAE-Zn coating could be ascribed as follows: as we all know, the nano-scale structures are vulnerable to being damaged under external forces, which results in the loss of superhydrophobicity. However, as the stressed parts of the armor-like structure, the large-scale micro-plates can effectively resist external force and protect the superhydrophobic nano-plates from the external impact.^40^ More importantly, the vertically aligned plate-like frame as stressed part not only distributed the mechanical pressure but also maintained the contact area between the surface and liquid basically unchanged. Consequently, even if the armored structure subjects to a certain degree of abrasion, their unique micro-nano plate-like array can still provide enough roughness and low contact area between the surface and liquid for the EAE-Zn coating to maintain the superhydrophobicity.

### Corrosion resistance

**Fig. 7:**
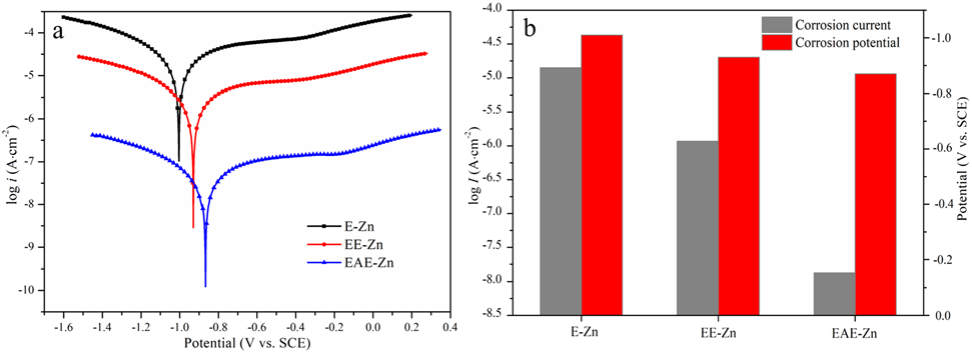
The Tafel curves (a) and the corrosion potential and corrosion current density (b) of E-Zn, EE-Zn and EAE-Zn coatings in 3.5 wt% NaCl aqueous solution.

Considering that the superhydrophobic surface can efficiently block the diffusion of corrosive medium to metal surface, the corrosion resistance of EAE-Zn coating in neutral 3.5 wt% NaCl solution were evaluated by potentiodynamic polarization technique. For comparison, the Tafel tests of E-Zn coating and EE-Zn coating were also performed and recorded. The corrosion potential (*E*_corr_) and corrosion current density (*i*_corr_) of all the specimens were obtained by Tafel extrapolation method and shown in Fig. 7b. As shown in Fig. 7a, the flower-like E-Zn coating had the highest corrosion current density (*i*_corr_ = 1.42 × 10^−5^ A cm^−2^) and the lowest corrosion potential (*E*_corr_ = −1.01 V). When the nano-plates were deposited sparsely on the flower-like structures, the *E*_corr_ of the obtained EE-Zn coating slightly shifted to the positive direction and the *i*_corr_ of EE-Zn coating reduced to 1.17 × 10^−6^ A cm^−2^. Notably, as the side surfaces of micro-plates were covered completely by the crisscrossing nano-plates, the *i*_corr_ of EAE-Zn coating was as low as 1.34 × 10^−8^ A cm^−2^, which was approximately 3 and 2 orders of magnitude lower than that of E-Zn and EE-Zn coatings, respectively. Obviously, the corrosion resistance of EAE-Zn coating is better than that of other Zn-based superhydrophobic coatings reported in the literature.^51–54^ Therefore, it could be concluded that the superhydrophobic EAE-Zn coating with remarkable anti-corrosion performance could be attributed to the formation of stable air cushion entrapped among the micro-nano plates, which could efficiently hinder corrosive medium to penetrate into the as-prepared coating.

**Fig. 8:**
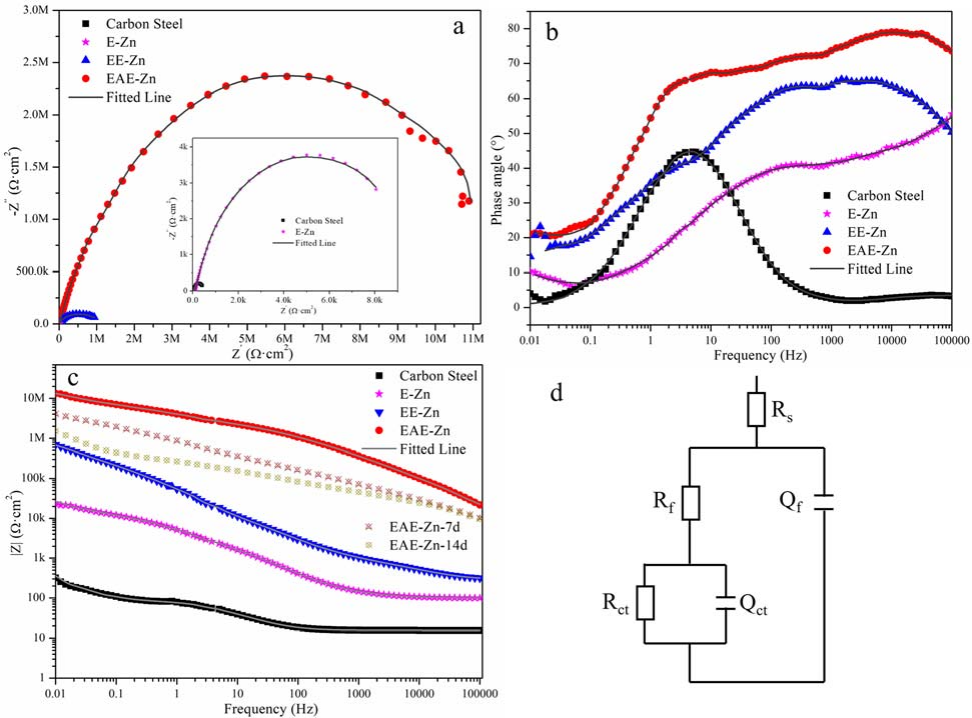
Nyquist plots (a) Bode plots of negative phase angle *vs.* frequency (b), Bode plots of |*Z*| value *vs.* frequency (c) and the equivalent circuit model (d) of E-Zn, EE-Zn and EAE-Zn coatings in 3.5 wt% NaCl solution.

In order to better analyze the corrosion behavior, the electrochemical impedance spectra (EIS) were implemented on E-Zn, EE-Zn and EAE-Zn coating in 3.5% NaCl solution. The radius of the capacitive loop and low-frequency impedance modulus |*Z*|_0.01 Hz_ value are usually introduced to evaluate the corrosion resistance of coatings. It could be seen that three coating all featured with only one capacitive loop with different radius (Fig. 8a). The radius of the capacitive loop from E-Zn, EE-Zn and EAE-Zn coating increased significantly in sequence, indicating the superhydrophobic EAE-Zn coating held the highest corrosion resistance. As shown in Fig. 8b, the phase angle value of EAE-Zn coating at the high-frequency region was much bigger and wider than that of E-Zn and EE-Zn coatings. As shown in Fig. 8c, the |*Z*|_0.01 Hz_ values of E-Zn coating and EE-Zn coating were 2.12 × 10^4^ and 8.55 × 10^5^ Ω cm^2^, respectively. However, the |*Z*|_0.01 Hz_ value of EAE-Zn coating was as high as 1.72 × 10^7^ Ω cm^2^, which was about 3 and 2 orders of magnitude greater than that of E-Zn and EE-Zn coating, respectively. The results demonstrated that the armor-like superhydrophobic coating presented excellent chemical inertness and impermeability to chloride ions, which was in agreement with the Tafel results.

Subsequently, the EIS data was fitted by the Zview software and related fitting parameters to accurately assess the electrochemical corrosion performance, as displayed in Table 3. An equivalent circuit model with two-time constants was adopted to simulate the corrosion behaviors of E-Zn, EE-Zn and EAE-Zn coating. In the circuit model, *R*_s_, *R*_f_ and *R*_ct_ represent the solution resistance, the Zn-based coating resistance and the charge transfer resistance of the double layer, respectively. The *Q*_dl_ and *Q*_f_ are the constant phase element, which represent the double layer capacitance and Zn-based coating capacitance, respectively. The value of *R*_ct_ is one of the most important indicators used in evaluating the corrosion resistance of coating. As can be seen from Table 3, the *R*_ct_ of superhydrophobic EAE-Zn coating was more than 3 and 2 orders of magnitude higher than that of the E-Zn and EE-Zn coating, respectively. Besides, the *Q*_dl_ value of the EAE-Zn coating was extremely small compared with E-Zn and EE-Zn coating, indicating that the charge transfer process was difficult for the superhydrophobic EAE-Zn coating. It was obvious that the *Q*_f_ value of superhydrophobic EAE-Zn coating was the lowest, and the *n*_f_ value was nearly 1, illustrating that the armor-like hierarchical structure possessed outstanding chemical inertness and resistance to chloride permeability.^4,55^

**Table 3:** The fitted electrochemical parameters of the EIS results for E-Zn, EE-Zn and EAE-Zn coatings

Specimens	*R*_s_ Ω^−1^ cm^−2^	*Q*_f_		*R*_f_ Ω cm^2^	*Q*_dl_		*R*_ct_ Ω^−1^ cm^−2^
		*Y*_c_ (Ω^−1^ cm^−2^ s^*n*^)	*n*		*Y*_dl_ (Ω^−1^ cm^−2^ s^*n*^)	*n*	
E-Zn	143	1.12 × 10^−5^	0.87	1.06 × 10^3^	1.57 × 10^−5^	0.74	8.37 × 10^3^
EE-Zn	147	2.07 × 10^−6^	0.89	4.39 × 10^4^	4.84 × 10^−6^	0.71	9.31 × 10^5^
EAE-Zn	151	1.81 × 10^−7^	0.92	6.91 × 10^5^	2.72 × 10^−7^	0.70	1.12 × 10^7^

Furthermore, the results of chemical stability test indicated that the armor-like EAE-Zn coating could maintain satisfied superhydrophobicity in 3.5% NaCl solution for long duration. To evaluate the long term anti-corrosion property, the Bode plots of EAE-Zn superhydrophobic coating immersed in 3.5 wt% NaCl solution for 7 and 16 days was obtained. As presented in Fig. 8c, the |*Z*|_0.01 Hz_ value of EAE-Zn coating only presented a slight decline within the initial 7 days' immersion. More importantly, despite the |*Z*|_0.01 Hz_ value of EAE-Zn coating decreased greatly when the immersion time increased to 14 days, it was still higher than that of E-Zn and EE-Zn coatings. The above results suggested that the superhydrophobic EAE-Zn coating with armor-like hierarchical structure presented a better long-term anti-corrosion performance.

## Conclusions

A robust hierarchical superhydrophobic Zn coating was fabricated by two-step electrodeposition accompanied with an indispensable intermediate activation. After the activation treatment, the deposition process changed from the mainly growth of micro Zn plates to the formation/growth of nano plates owing to abundant active sites formed on the side surfaces of micro Zn plates. The results indicated that the surface hierarchical structure could be adjusted by introducing the activation treatment to change the deposition process. The armour-like Zn coating consisting of micro-plates and nano-plates (EAE-Zn) showed superior super-repellency to a variety of liquid drops such as distilled water, soda water, black tea, milk, coffee, juice and methyl orange. More importantly, the Zn coating with activation treatment showed superior super-repellency, corrosion resistance and mechanical-chemical stability than that without activation treatment. This indicated the vertically aligned micro-plates acting as ‘armour’ were effectively against the abrasion of fragile nano-structures. The strategy proposed in this work provided guidance for the construction of novel hierarchical structures with robust superhydrophobicity. The armour-like superhydrophobic Zn coating exhibits substantial application potential in fields such as corrosion protection of marine equipment and microfluidic devices.

## Author contributions

Mengqing Li: conceptualization, software, data curation, investigation, writing draft. Liangjun Song: software, data curation, validation. Yu Zheng: software, data curation, validation. Nuoran Liu: software, data curation, validation. Huizhu Yu: data curation, supervision, funding acquisition. Rencheng Jin: investigation, data curation, visualization. Tengfei Xiang: writing – review & editing, supervision, funding acquisition. Ruiqian Li: conceptualization, supervision, writing – review & editing, funding acquisition.

## Conflicts of interest

We declare that we have no known competing financial interests or personal relationships that could have appeared to influence the work reported in this paper.

## Data Availability

Data are available upon request from the authors.
